# Willingness to use female permanent contraception among married women and male partners´ support in Benue, Nigeria

**DOI:** 10.11604/pamj.2025.50.96.35622

**Published:** 2025-04-09

**Authors:** Babayemi Oluwaseun Olakunde, Ijeoma Uchenna Itanyi, Amaka Grace Ogidi, Imoter Uparegh, John Okpanachi Oko, Chima Ariel Onoka, Echezona Edozie Ezeanolue

**Affiliations:** 1 Center for Translation and Implementation Research, University of Nigeria, Nsukka, Enugu, Nigeria,; 2 Department of Population and Community Health, College of Public Health, University of North Texas Health Science Center, Fort Worth, Texas, United States of America,; 3 Department of Community Medicine, University of Nigeria Nsukka, Enugu, Nigeria,; 4 Dalla Lana School of Public Health, University of Toronto, Toronto, Canada,; 5 Caritas Nigeria, Abuja, Federal Capital Territory, Nigeria

**Keywords:** Female sterilization, tubal ligation, male factor, acceptability, intention, willingness, Nigeria

## Abstract

**Introduction:**

there is a paucity of community-based studies on the acceptability of female permanent contraception (FPC), particularly among men. We examined the willingness of married women to use FPC and their male partners to support its use in Nigeria.

**Methods:**

we conducted a cross-sectional study among pregnant women and their male partners who participated in the Healthy Beginning Initiative (HBI) program in Benue, Nigeria. The HBI was an integrated, feasible, and culturally adaptive platform for screening, linkage, and follow-up of pregnant women attending churches. Data on sociodemographic characteristics and reproductive intentions were collected from 10,168 pregnant women and 6,766 male partners independently through pre-tested semi-structured interviewer-administered questionnaires. We performed univariate and multivariate logistic regression analyses to examine factors associated with the willingness to use FPC among women and male partners to support the use of FPC. Our analysis was restricted to married participants.

**Results:**

of the 10,046 married women and 6,759 men included in this study, 80% and 87% indicated willingness to use and support the use of FPC, respectively. In the adjusted model, women with no formal, primary, and secondary education (vs tertiary education) and those with an income level of ≤ ₦20,000 (vs > ₦50,001) had significantly higher odds of willingness to use FPC, while women with no living children (vs ≥ 5) and 0-2 desired children (vs ≥ 5) had significantly lower odds of intending to use FPC. Except for no formal education and the number of living children, similar factors were associated with the willingness of married men to support the use of FPC.

**Conclusion:**

there was a high willingness to use or support the use of FPC among married women and men in this study. Increasing access to FPC services in this setting may improve its informed and voluntary uptake.

## Introduction

With an estimated population of over 200 million, Nigeria is among the ten largest countries in the world, and its population is projected to double its current size by 2050 [1]. On average, the total fertility rate is about five births per woman [2]. However, it is estimated that women in Nigeria have 0.5 more children than they want [2]. Although the demand for family planning in Nigeria is low, nearly 39% of the demand among married women is for limiting childbearing [2]; with the majority of the women with met need for family planning to limit childbearing using short-acting and traditional methods [3]. Female permanent contraception (FPC), a modern contraceptive method that permanently limits childbearing among women, accounts for less than two percent of the method mix among married women in Nigeria [2]. The use of less effective contraceptive methods by clients who want to stop childbearing contributes to the burden of unintended pregnancies [4] and the attendant health and socioeconomic consequences [5,6].

Female permanent contraception (FPC) is the most commonly used contraceptive method globally [7,8]. It is effective, safe, and has no side effects associated with hormonal contraceptive methods [9]. Because it is a one-off procedure, FPC has been described as a “convenient” contraceptive method as it eliminates the need for periodic clinic visits for family planning [10,11]. It also has non-contraceptive benefits such as reduced risk of ovarian cancer [12-14]. More importantly, it is one of the most cost-effective contraceptive methods, given its extended duration of protection at no added cost [15,16]. Despite its advantages, there is low uptake of FPC among the increasing number of women who want to stop childbearing in sub-Saharan Africa (SSA) [3].

The barriers affecting the use of FPC in SSA, including Nigeria, exist at multiple levels. However, access to the procedure remains one of the important limiting factors [17]. In many countries in SSA, a significant proportion of health facilities that provide family planning services do not offer FPC [18]. If available, studies in developing countries have reported willingness to use FPC, ranging from 18% to 84% among women of reproductive age [19-22]. Indeed, evidence suggests that making FPC services available and affordable may improve its uptake in SSA [23-26]. Given the patriarchal nature of many societies in SSA, male partners’ support has also been found to influence the choice of FPC among women who desire to stop childbearing [27,28].

While a few studies have reported on the acceptability of FPC among women attending antenatal care in Nigeria [22,29], there is a paucity of community-based studies, particularly among men. Insight into the acceptability of FPC can inform interventions to improve access to FPC services. Thus, in this study, we examined the willingness of women to use FPC and male partners to support its use in Benue State, Nigeria. We defined willingness to use FPC as women´s consideration for FPC to limit childbearing after achieving the number of desired children, and male support as the willingness to support wives´ decision to use FPC to limit childbearing after achieving the number of desired children.

## Methods

**Study design:** we conducted a cross-sectional study among a cohort of pregnant women and their male partners who participated in the Healthy Beginning Initiative (HBI) program in Benue State, North-Central Nigeria.

**Study setting:** the HBI theoretical framework for maternal child health interventions was supported by the National Institutes of Health [30] and implemented as a program by a United States President's Emergency Plan for AIDS Relief (PEPFAR)-supported implementing partner, Caritas Nigeria in Benue State, Nigeria. A total of 80 churches in 80 communities across 12 Local Government Areas (LGAs) in Benue State participated in this program from June 2016 to October 2018. Benue State has an estimated population of about six million [31] of which about 70% are farmers. The state has the second highest prevalence of HIV in Nigeria, at 4.9% [32]. Benue State has an estimated total fertility rate of 4.3 [2].

The HBI provides an integrated, feasible, and culturally adaptive platform for screening, linkage, and follow-up of pregnant women with an aim to identify, treat, and retain women in care through their pregnancy. Details of the HBI have been described elsewhere [33,34]. In brief, HBI consists of three components. 1) Prayer sessions: every Sunday, the priest announced that pregnant women and their male partners come out for prayers. He prayed for a healthy pregnancy, safe delivery and encouraged pregnant women to seek antenatal care at a health facility; 2) baby showers were organized as a reception and health fair in churches during which health education on early antenatal care, importance of the integrated screening tests for pregnant women, good nutrition, skilled birth attendance, baby feeding options and immunizations were provided. On-site integrated laboratory testing for HIV, Hep B virus, and haemoglobin genotype was conducted for pregnant women and their male partners. Male partners received a “mama pack” to present to their female partners as an expression of love and support during the pregnancy; 3) baby receptions were organized six to eight weeks after delivery, for women who participated in the baby shower to be received in the church. The women completed a post-delivery questionnaire, which provided an opportunity to ascertain the place of delivery, pregnancy outcome, baby feeding option adopted, and immunization. This also offered an opportunity for post-delivery linkage to care for women and their babies who needed care.

**Participants:** participants were eligible to participate in this study if they were pregnant women or male partners of pregnant women, and participated in the baby shower program delivered in churches in Benue State. This was a whole population study of participants in the HBI, so no sampling technique was used.

**Data collection:** trained program assistants who had a minimum of a Bachelor´s degree administered the questionnaires. Data was collected from all participants on sociodemographic characteristics, including age, sex, marital status, highest level of education, occupation, monthly income, languages spoken, and distance to health facility, using pre-tested semi-structured interviewer-administered questionnaires. On reproductive health, information on current family size and intended family size was obtained from the respondents. In addition to other reproductive health-related questions, females were asked about their intent for FPC. The specific question asked was: “If God grants you your desired number of children, will you consider tying your womb so that even though you continue meeting with your husband, you will not be scared of having more children mistakenly?”. The responses were “yes” or “no”. Male partners were also asked about their willingness to support their spouses in using permanent contraception as follows: “If God grants you your desired number of children, will you support your wife to tie her womb so that even when you continue to meet with her, there will not be any fear of having more children mistakenly?”. Male partners and their wives were interviewed separately.

**Ethical considerations:** ethical approval was obtained from the Health Research Ethics Committee of the University of Nigeria Teaching Hospital, Enugu. Written consent was obtained from the study participants.

**Data analysis:** we restricted our analysis to only married participants because the survey question for the analysis asked about male support of their wives´ FPC. We performed descriptive analysis to summarize the data and reported the willingness to use or support the use of FPC among married women and men, respectively in percentages. We performed logistic regression analyses to examine the factors associated with the willingness to use FPC among women and the willingness of male partners to support their spouses to use FPC. Factors that were significant in the univariate logistic regression analyses were included in the multivariate logistic regression, after checking for multicollinearity. We reported crude odds ratio (OR) and adjusted OR (aOR) and their 95% confidence intervals (CI) for the factors included in the univariate and multivariate models, respectively. P-value < 0.05 was considered statistically significant. The data analysis was performed with SPSS version 25.

## Results

**Characteristics of the participants:** of the 10,168 pregnant women and 6,766 male partners who participated in the HBI, 10,046 married pregnant women and 6,759 married men were included in this study. Table 1 shows the characteristics of the respondents. The majority of the women were younger than 35 years and earned ₦20,000 ($65) or less monthly. About 82% had at least primary education, while 24% had no living children. Approximately 52% of the women desired at least five children. About 47% of the men were between the ages of 25 and 34 years. Over 90% had at least primary education, and 83% earned ₦20,000 ($65) or less. Nearly 20% reported not having a child, while 58% desired having five or more children. Approximately 80% of women indicated willingness to use FPC, while 87% of men indicated that they were willing to support their spouses to use FPC when they achieved the desired family size (Table 1).

**Table 1:** characteristics of respondents and willingness to use or support the use of female permanent contraception among married female and male participants of HBI, Benue, Nigeria

Characteristics	Women N* (%)	Men N**(%)
**Age**		
≤ 24	5435 (54.1)	1075 (15.9)
25-34	4065 (40.5)	3204 (47.4)
≥ 35	546 (5.4)	2480 (36.7)
**Educational attainment**	**N=10043**	**N=6757**
No formal education	1761 (17.7)	381 (5.6)
Primary	2959 (29.4)	1240 (18.4)
Secondary	4676 (46.5)	3921 (58.0)
Tertiary	647 (6.4)	1215 (18.0)
Income***	**N=10044**	**N=6754**
≤ ₦20,000	9264 (92.2)	5589 (82.8)
₦20,001- ₦50,000	604 (6.0)	815 (12.1)
≥ ₦50,001	176 (1.8)	350 (5.2)
**Number of living children**		**N=6758**
0	2368 (23.6)	1327 (19.6)
1-2	4275 (42.5)	2560 (37.9)
3-4	2338 (23.3)	1647 (24.4)
≥ 5	1065 (10.7)	1224 (18.1)
**Number of male children**		**N=6758**
0	4268 (42.5)	2440 (36.1)
1-2	4794 (47.7)	3292 (48.7)
3-4	872 (8.7)	778 (11.5)
≥ 5	112 (1.1)	248 (3.7)
**Number of female children**		
0	4268 (42.5)	2407 (35.6)
1-2	4563 (45.4)	3135 (46.4)
3-4	1059 (10.5)	875 (12.9)
≥ 5	156 (1.6)	342 (5.1)
**Number of desired children**		**N=6754**
0-2	197 (2.0)	105 (1.6)
3-4	4653 (46.3)	2712 (40.2)
≥ 5	5196 (51.7)	3937 (58.3)
**Distance to health facility**		
≤ 5 Km	7481 (74.5)	5258 (77.8)
6-10 Km	2235 (22.2)	1335 (19.8)
≥11 Km	330 (3.3)	166 (2.5)
**Willingness to use/support the use of female permanent contraception**		
No	1984 (19.7)	870 (12.9)
Yes	8062 (80.3)	5889 (87.1)

* N: 10,046 except where stated; **N: 6759 except where stated; ***: 305 Naira (₦) = 1 USD ($)

**Factors associated with the willingness of women to use FPC:** among the women, educational attainment, income, number of living children, and the desired number of children were significantly associated with the willingness to use FPC in the univariate regression analyses, and these factors remained statistically significant in the adjusted model (Table 2). The odds of willingness to use FPC in women who had secondary, primary, or no formal education were nearly two times that of women who had tertiary education (Table 2). Compared to women who earned at least ₦50,001 ($164), those who earned ₦20,000 ($65) or less had higher odds of willingness to use FPC (aOR=1.8, 95%CI=1.33-2.55). Women with no living children (aOR=0.7, 95%CI=0.54-0.82) and those who desired less than three children (aOR=0.5, 95%CI=0.38-0.71) had significantly lower odds of willingness to use FPC compared to women with at least five living children and those who desired at least five children, respectively (Table 2).

**Table 2:** factors associated with willingness to use or support the use of female permanent contraception among married female and male participants of HBI, Benue, Nigeria

Characteristics	Women		Men	
	OR (95%CI)	aOR (95%CI)	OR (95%CI)	aOR (95%CI)
**Age (ref: ≥ 35)**				
≤ 24	0.8 (0.65-1.07)		0.8 (0.68-1.02)	
25-34	0.9 (0.70-1.13)		1.1 (0.98-1.34)	
**Educational attainment (ref: tertiary)**				
No formal education	1.8 (1.49-2.26) ***	1.6 (1.29-1.98) ***	1.2 (0.87-1.68)	1.0 (0.75-1.47)
Primary	1.9 (1.57-2.31) ***	1.7 (1.39-2.07) ***	1.5 (1.15-1.83) **	1.3 (1.01-1.63) *
Secondary	1.7 (1.38-1.99) ***	1.6 (1.31-1.90) ***	1.4 (1.15-1.65) **	1.2 (1.03-1.49) *
**Income (ref: ≥ ₦50,001)**				
≤ ₦20,000	1.9 (1.39-2.64) ***	1.8 (1.33-2.55) ***	2.0 (1.54-2.63) ***	1.9 (1.44-2.51) ***
₦20,001- ₦50,000	1.4 (0.94-1.97)	1.3 (0.92-1.94)	1.3 (0.96-1.80)	1.3 (0.93-1.76)
**Distance to health facility (ref: ≤ 5 Km)**				
6-10Km	0.9 (0.71-1.21)		1.1 (0.68-1.75)	
≥11Km	1.2 (0.94-1.20)		1.0 (0.86-1.23)	
**Number of living children (ref: ≥ 5)**				
0	0.6 (0.51-0.75) ***	0.7 (0.54-0.82) ***	0.9 (0.68-1.07)	
1-2	0.8 (0.68-0.97) *	0.9 (0.71-1.05)	1.0 (0.85-1.27)	
3-4	0.9 (0.77-1.14)	1.0 (0.78-1.17)	1.1 (0.88-1.38)	
**Number of male children (ref: ≥ 5)**				
0	0.7 (0.41-1.14)		0.8 (0.52-1.16)	
1-2	0.8 (0.51-1.41)		1.0 (0.70-1.56)	
3-4	0.9 (0.54-1.57)		0.8 (0.49-1.18)	
**Number of female children (ref: ≥ 5)**				
0	0.7 (0.43-1.02)		1.0 (0.69-1.34)	
1-2	0.9 (0.55-1.32)		1.0 (0.76-1.47)	
3-4	1.0 (0.64-1.59)		1.1 (0.78-1.65)	
**Number of desired children (ref: ≥ 5)**				
0-2	0.5 (0.34-0.62) ***	0.5 (0.38-0.71) ***	0.6 (0.35-0.92) *	0.6 (0.34-0.90) *
3-4	0.9 (0.78-0.96) **	1.0 (0.87-1.08)	0.9 (0.80-1.07)	0.9 (0.80-1.08)

* : p<0.05; **: p<0.01; ***: p<0.001; blank cell indicates factor was not significant at the univariate level

**Factors associated with the willingness of men to support their female partners to use FPC:** similarly, in men, the willingness to support FPC was associated with level of education, income, and the desired number of children in the adjusted model (Table 2). The odds of willingness to support FPC in men who had primary and secondary education were 1.3 and 1.2 times that of men with tertiary education (Table 2). Also, the odds of willingness to support FPC in men who earned ≤ ₦20,000 ($65) were 1.9 times that of men who earned at least ₦50,001 ($164) (Table 2). Compared to men who desired five or more children, those who desired fewer than three children had lower odds of willingness to support FPC (aOR=0.6, 95%CI=0.34-0.90) (Table 2).

## Discussion

In this study, we examined the willingness to use FPC among married pregnant women and the willingness of married men to support their spouses to use FPC in Benue State, Nigeria. Eighty percent of the women in our study indicated a willingness to use FPC when they achieve their desired family size. Among men, nearly 9 in 10 expressed willingness to support their spouses to use FPC. In the adjusted regression analyses, educational attainment, income, number of living children, and the desired number of children were statistically associated with the willingness to use FPC among women, while in men, educational attainment, income, and the desired number of children were the significant factors.

The high proportion of women and men who reported their willingness to use or support the use of FPC, respectively, in this study may be a result of good awareness of this method or the perceived benefits from the description of the method in our survey question. In addition to limited access, uptake of FPC in SSA is affected by poor knowledge, including myths and misconceptions about the method [17]. Concerns that the procedure is surgical and may be associated with complications, also prevent women who desire to limit birth from using FPC [22,35,36]. Consistent with our finding, a similar magnitude of intention to use FPC (84%) was reported among married women in rural areas in Nepal [19]. Our finding, however, was much higher than another study in Nigeria by Enyindah *et al*. which reported that only 18% of 383 women attending antenatal care in South-South Nigeria would consider using FPC in the future [22]. A lower intention to use FPC (20%) was also reported among married women in Ethiopia, where only about 50% of the respondents were aware of its advantages [21].

Even when it is desired, lack of support from male partners may hinder women´s use of FPC [37,38]. In Nigeria, male partners play an influential role in decision-making regarding utilization of contraception [39,40], thus it was encouraging to find that a high proportion of male partners were willing to support FPC in our study. This finding suggests that among the population we studied, if available and accessible, male factor may not be a barrier to improving voluntary and informed uptake of FPC. Interestingly, the proportion of male partners willing to support the use of FPC was higher than the proportion of women willing to use FPC. Perhaps, since men will not be bearing the burden of undergoing the procedure, they may be more willing to support FPC [41].

Consistent with a previous study in Uganda [20], we found lower willingness to use FPC among women with no living children. High demand for children is often found across different societies in Nigeria, largely for cultural and socioeconomic reasons [42-44]. Thus, given the permanent nature of the procedure, it may not be appealing to women without children. We had expected that participants with a desire for a small family size would be more willing to use FPC, however, the result showed the reverse. It is possible that those who desire a small family size may not see the need for permanently limiting childbearing as they do not plan to have many children.

As observed in our findings, poorer and less educated women and men were significantly more willing to use or support the use of FPC. These categories of people may have health and economic challenges; which have been identified as some of the motivating factors for permanently limiting childbearing in SSA [17]. Despite their willingness, the use of FPC among the poor in Nigeria may be inhibited by the cost of the procedure [45], if it is not subsidized or provided free of charge. Although the FPC is cost-effective in the long run, the cost of undergoing the procedure may be higher than other reversible methods [45].

One of the strengths of this study lies in the large sample size of the women and men included in the study. To our knowledge, this is the first study to report on the willingness to support FPC among male partners in Nigeria.

**Limitations:** our study population was limited to married pregnant women and men who participated in the HBI Program in Benue State, with a potential for selection bias; thus the results cannot be generalized to non-participants of the HBI, unmarried persons in Benue State, and other settings in Nigeria. While we provided a description of FPC to the respondents, we did not assess or control for their knowledge of the method. We chose simplified binary responses (yes/no) to the questions that assessed their willingness to use or support the use of FPC, based on our understanding of the context, which was predominantly rural. While this could oversimplify our assessment of their attitudes, it limits the potential for misclassification bias by directly assessing their willingness in a way that they would understand clearly. Additionally, religion, an important factor that may influence the willingness to use FPC, was not considered in this study because the HBI program was only implemented in churches. We recommend more studies to explore the willingness to use FPC and determinants in other settings in Nigeria.

## Conclusion

Our findings showed high willingness to use FPC among married pregnant women and even higher willingness among married men to support their spouses to use the permanent contraceptive method in Benue, Nigeria. These findings suggest that married clients seeking to limit childbearing in our study setting may not be averse to FPC. While willingness to use may not necessarily translate to eventual use, increasing access to FPC services in this setting may improve its informed and voluntary uptake.

### What is known about this topic

- Female permanent contraception is the most commonly used contraceptive method globally;
- Despite its advantages, there is low uptake of female permanent contraception among women who want to stop childbearing in sub-Saharan Africa;
- Poor access to female permanent contraception remains one of the important barriers to its use in sub-Saharan Africa.

### What this study adds

- There is a high willingness among married pregnant women in Nigeria to use female permanent contraception to limit childbearing;
- There is even a higher willingness among married men to support their spouses to use female permanent contraception;
- The high willingness to use and support the use of female permanent contraception by pregnant women and their male partners, respectively, highlights the need to make its services accessible to pregnant women in Nigeria.
